# Bilateral Auricular Blastomycosis-like Pyoderma: A Rare Presentation Histologically Misinterpreted as Squamous Cell Carcinoma

**DOI:** 10.3390/dermatopathology13020016

**Published:** 2026-04-01

**Authors:** Nazario Pesce, Giorgia Di Marco, Giorgio Stabile, Antonio Podo Brunetti, Alessandro Russo, Stefania Guida, Rongioletti Franco

**Affiliations:** 1 Dermatology Clinic, IRCCS San Raffaele Hospital, 20132 Milan, Italy; pesce.nazario@hsr.it (N.P.); stabile.giorgio@hsr.it (G.S.); podobrunetti.antonio@hsr.it (A.P.B.); russo.alessandro@hsr.it (A.R.); guida.stefania@hsr.it (S.G.); rongioletti.franco@hsr.it (R.F.); 2 School of Medicine, Vita-Salute San Raffaele University, 20132 Milan, Italy

**Keywords:** blastomycosis-like pyoderma, pyodermitis vegetans of Azua, hyperinflammatory proliferative pyoderma, pyoderma vegetans, squamous cell carcinoma, ulcerative colitis, misdiagnosis, systematic review

## Abstract

Blastomycosis-like pyoderma (BLP) is a rare chronic inflammatory dermatosis characterized by exuberant vegetative and verrucous plaques, most frequently associated with bacterial colonization, particularly *Staphylococcus aureus*. Owing to its striking clinical and histopathological resemblance to squamous cell carcinoma (SCC) and other granulomatous or hyperplastic dermatoses, BLP represents a well-recognized diagnostic pitfall, often leading to delayed diagnosis or unnecessary surgical management. We report an unusual case of bilateral auricular BLP in a 58-year-old apparently immunocompetent woman, initially misdiagnosed as SCC. Comprehensive clinicopathological reassessment revealed pseudoepitheliomatous hyperplasia, intraepidermal neutrophilic microabscesses, and a dense mixed inflammatory infiltrate, findings consistent with a reactive rather than neoplastic process. Microbiological cultures confirmed *Staphylococcus aureus*, supporting the final diagnosis of BLP and guiding effective antimicrobial therapy. To better contextualize this rare presentation, we reviewed all previously reported cases of BLP, summarizing available clinical, histopathological, microbiological, and therapeutic data. This case further raises the possibility of an association between BLP and systemic inflammatory conditions, as the patient subsequently developed severe colitis, highlighting the potential role of immune dysregulation and the gut–skin axis in disease pathogenesis or a possible temporal association, without allowing causal inference. Beyond inflammatory bowel disease, blastomycosis-like pyoderma has been reported in association with a variety of systemic and immune-mediated conditions, including diabetes mellitus, hematologic malignancies, HIV infection, chronic renal failure, autoimmune disorders, and prolonged immunosuppressive therapies. These associations support the concept that BLP represents a hyperinflammatory reaction pattern occurring in the setting of altered immune surveillance rather than a purely infectious disease. Accurate recognition and management of BLP require careful integration of clinical features, histological findings, and microbiological results. Increased awareness of its diverse presentations is essential to avoid misdiagnosis and to ensure appropriate, conservative treatment.

## Simple Summary

Blastomycosis-like pyoderma is a rare chronic inflammatory skin condition characterized by thick, vegetating or wart-like plaques that arise as an exaggerated response to persistent bacterial stimulation in susceptible patients. Because its clinical presentation and histological features can closely resemble skin cancer, it is frequently misdiagnosed, sometimes leading to unnecessary aggressive treatments. In this article, we report an unusual case of blastomycosis-like pyoderma involving both ears of a middle-aged woman with no known immune disorders, initially diagnosed as squamous cell carcinoma. Re-evaluation of the skin biopsy, together with microbiological investigations, demonstrated an inflammatory process associated with bacterial infection rather than malignancy. Culture-guided antibiotic therapy resulted in marked clinical improvement. This case underscores the importance of integrating clinical, histological, and microbiological findings to achieve an accurate diagnosis and to promote appropriate, conservative management in dermatology and dermatopathology.

## 1. Introduction

Blastomycosis-like pyoderma (BLP) is a rare chronic vegetative inflammatory dermatosis characterized by exuberant verrucous or pustular plaques, most often arising in association with bacterial infection.

Although the terms blastomycosis-like pyoderma and pyoderma vegetans have historically been used interchangeably in the literature, this equivalence is not universally accepted. Several authors propose a conceptual distinction, whereby blastomycosis-like pyoderma is regarded as a hyperplastic cutaneous reaction to persistent bacterial stimulation, whereas pyoderma vegetans is more closely associated with inflammatory bowel disease and is often considered within the spectrum of neutrophilic dermatoses. In this context, blastomycosis-like pyoderma is typically characterized by prominent pseudoepitheliomatous hyperplasia and bacterial colonization, while pyoderma vegetans more frequently shows a systemic inflammatory background.

Although *Staphylococcus aureus* is the microorganism most frequently implicated, BLP is currently regarded not as a primary infectious disease but as a reactive cutaneous response to persistent microbial stimulation. Rather than resulting from direct tissue invasion, the disease appears to reflect an exaggerated host inflammatory response, driven by sustained antigenic exposure and dysregulated cutaneous immunity. Historically, the condition has been described predominantly in patients with some degree of immune compromise, including diabetes mellitus, hematologic malignancies, chronic renal failure, HIV infection, or prolonged immunosuppressive therapy. However, increasing numbers of cases reported in apparently immunocompetent individuals suggest that overt systemic immunosuppression is not a prerequisite for disease development and that subtle alterations of immune surveillance or skin barrier function may play a critical role.

One of the major challenges in the diagnosis of BLP lies in its striking clinical and histopathological resemblance to a wide spectrum of dermatological disorders. In particular, BLP may closely mimic deep fungal infections, chronic inflammatory dermatoses, and, most importantly, squamous cell carcinoma (SCC). Clinically, the presence of vegetative, ulcerated, or verrucous plaques with raised borders and purulent discharge may strongly suggest a malignant or infectious etiology, especially when lesions are localized to sun-exposed or chronically irritated areas. Histopathologically, the prominent pseudoepitheliomatous hyperplasia, associated with marked acanthosis and dense neutrophilic inflammation, can be easily misinterpreted as a neoplastic epithelial proliferation, particularly in limited or superficial biopsy specimens that do not capture the full architectural context of the lesion.

We report the case of a 58-year-old apparently immunocompetent woman presenting with a rare bilateral auricular manifestation of blastomycosis-like pyoderma, initially misdiagnosed as squamous cell carcinoma. Auricular involvement is uncommon in BLP, and bilateral presentation is exceptionally rare, further contributing to diagnostic uncertainty and reinforcing the potential for misclassification as a malignant process. The absence of overt immunosuppression in our patient challenges the traditional view of BLP as a condition restricted to immunocompromised hosts and supports the growing recognition that BLP may also occur in individuals with subtle or unrecognized immune dysregulation.

Current knowledge of blastomycosis-like pyoderma is largely derived from isolated case reports and small case series, limiting comprehensive understanding of its epidemiology, clinical spectrum, histopathological features, and optimal management strategies. The rarity of the condition and its heterogeneous presentation have hindered the development of standardized diagnostic and therapeutic approaches. Systematic consolidation of published cases is therefore essential to identify recurrent patterns, diagnostic pitfalls, and therapeutic outcomes. In this report, we present an unusual case of bilateral auricular BLP and contextualize our findings through a comprehensive review of previously reported cases. The rare anatomical distribution, initial misdiagnosis as SCC, and subsequent association with systemic inflammatory disease underscore the importance of maintaining a high index of suspicion and performing careful clinicopathological correlation when evaluating vegetative or hyperplastic dermatoses.

## 2. Materials and Methods

A narrative systematic review was conducted to integrate the present case into the broader context of published reports on blastomycosis-like pyoderma (BLP). A comprehensive search of the PubMed database was carried out using the single keyword “Blastomycosis-like pyoderma,” with no restrictions on publication date, language, or article type. This search yielded 37 records published between 1949 and 2024, including case reports, small case series, and review articles. Two reviewers (N.P. and G.D.M.) independently screened titles and abstracts, and full texts were examined when the diagnosis of BLP or a closely related entity—such as vegetative pyoderma, pyodermitis vegetans, or hyperinflammatory vegetative pyoderma consistent with BLP—was clearly documented.

For each eligible study, we extracted demographic information, anatomical distribution and morphology of lesions, diagnostic latency, histopathological features, microbiological findings, therapeutic strategies, and clinical outcomes. All extracted data were synthesized descriptively, and percentages were calculated when feasible. The findings of this systematic review were used to situate our case within the existing body of literature and to highlight recurring trends in presentation, diagnostic challenges, and therapeutic management across reported cases.

## 3. Case Report

A 58-year-old Caucasian woman with no known underlying medical conditions or history of immunosuppression presented with progressively enlarging granulomatous lesions involving both auricles, which had been present for approximately three months. The patient denied systemic symptoms, including fever, weight loss, night sweats, or gastrointestinal complaints at the time of dermatological evaluation. According to the patient’s history, the auricular lesions developed almost simultaneously on both ears, without a clear initial unilateral onset. She reported no recent trauma, insect bites, or prior dermatologic conditions affecting the ears.

Initial punch biopsies performed at another institution were interpreted as folliculitis and perifolliculitis. However, due to progressive enlargement and persistence of the lesions, a subsequent histopathological examination of a unilateral auricular lesion raised suspicion for squamous cell carcinoma (SCC). This interpretation was based on the presence of irregular epidermal hyperplasia, keratin-filled cystic structures, focal mild keratinocyte atypia, and a prominent inflammatory infiltrate. Given the unusual bilateral presentation, the absence of significant cytological atypia, and the patient’s otherwise unremarkable medical history, a second opinion was requested, and the patient was referred to our clinic for further evaluation.

**Figure 1:**
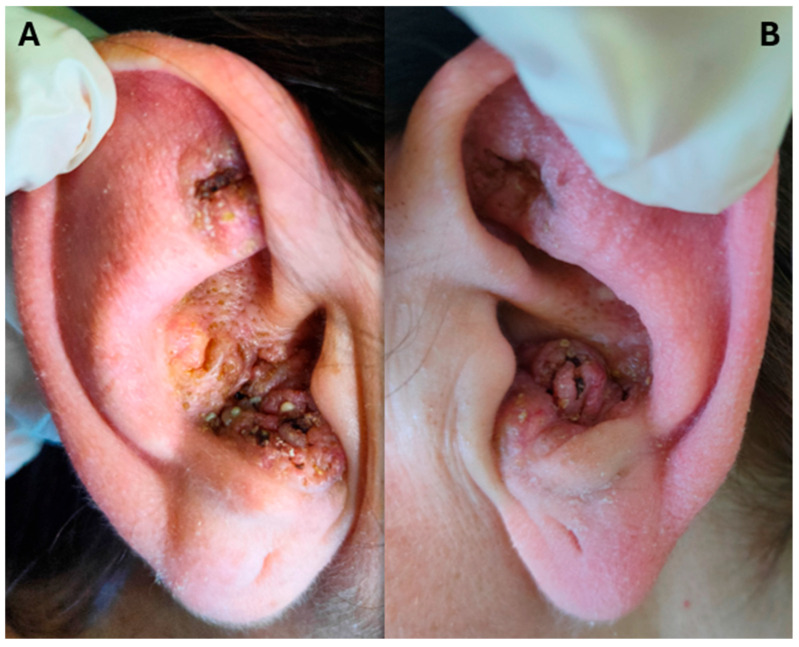
(**A**) Right ear at first clinical observation. (**B**) Left ear at first clinical observation.

On clinical examination, both auricles displayed multilobulated, vegetative, and pustular plaques with raised and irregular borders, central erosions, and multiple pustules of varying size distributed across the helix and antihelix (Figure 1). The lesions were moderately tender on palpation but non-bleeding. No regional lymphadenopathy was detected, and general physical examination was unremarkable. Laboratory investigations, including complete blood count and basic inflammatory markers, were within normal limits.

**Figure 2:**
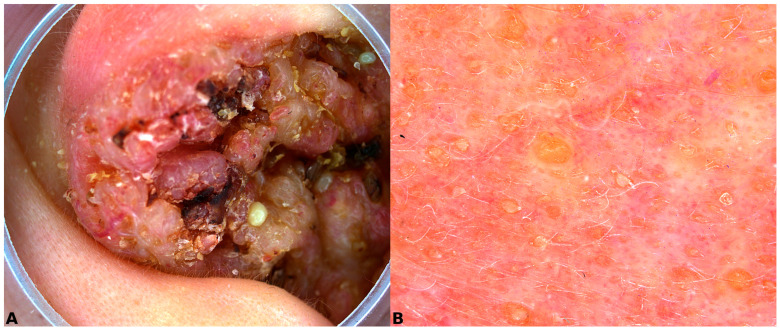
Dermoscopy of lesion. Lesion with verruciform aspects on an erythematous-yellowish background with keratinous follicular plugs. (**A**) 10× magnification; (**B**) 20× magnification.

Dermoscopy revealed a verruciform surface pattern set on an erythematous-yellowish background, with prominent keratinous follicular plugs and areas corresponding to pustular structures (Figure 2). Although these findings were not specific, they supported a hyperkeratotic and inflammatory rather than frankly neoplastic process.

**Figure 3:**
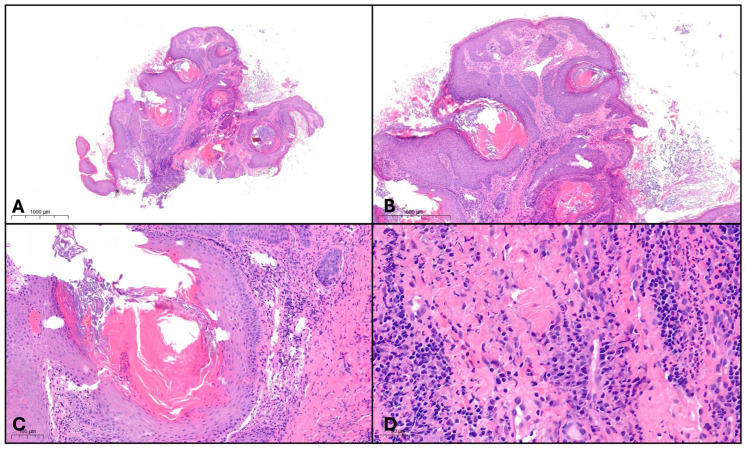
Histology of BLP. Hematoxylin and eosin stained sections. (**A**) Irregular exophytic hyperplasia of the epidermis with follicular ectasia and exacerbated dermal inflammation; 10× magnification. (**B**) Pseudoepitheliomatous epidermal hyperplasia with areas of hyperkeratosis and acanthosis; 20× magnification. (**C**) Dilated and keratin-plugged hypertrophic follicular infundibulum; 100× magnification. (**D**) Chronic mixed-type inflammatory infiltrate; 200× magnification.

A new incisional biopsy was performed, accompanied by bacterial and fungal cultures. Histopathological examination demonstrated marked pseudoepitheliomatous hyperplasia with overlying hyperkeratosis, intraepidermal and follicular neutrophilic microabscesses, and a dense chronic inflammatory infiltrate composed of lymphocytes, histiocytes, neutrophils, and scattered eosinophils (Figure 3). No significant cytological atypia, abnormal mitotic figures, or invasive growth into the dermis were identified. Special stains for fungi and mycobacteria were negative. Clinicopathological correlation favored a reactive pseudoepitheliomatous inflammatory lesion rather than malignancy. Bacterial cultures yielded *Staphylococcus aureus*, supporting the diagnosis of blastomycosis-like pyoderma.

Based on these findings, treatment was initiated with intramuscular ceftriaxone, oral doxycycline, and topical fusidic acid combined with betamethasone, tailored according to antibiotic susceptibility testing. Progressive clinical improvement was observed over the following weeks, with reduction in inflammation, pustulation, and lesion size.

Approximately one month after initiation of dermatological treatment, the patient developed acute abdominal pain and signs of bowel obstruction, necessitating emergency laparotomy. Surgical exploration revealed sigmoid bowel obstruction with hemorrhagic necrosis and marked granulocytic inflammation, without histological evidence of malignancy. The findings were considered consistent with acute colitis, although a definitive diagnosis of ulcerative colitis could not be established. The postoperative course was complicated by the development of a laparocele requiring surgical repair, followed by left colon perforation that necessitated hemicolectomy.

**Figure 4:**
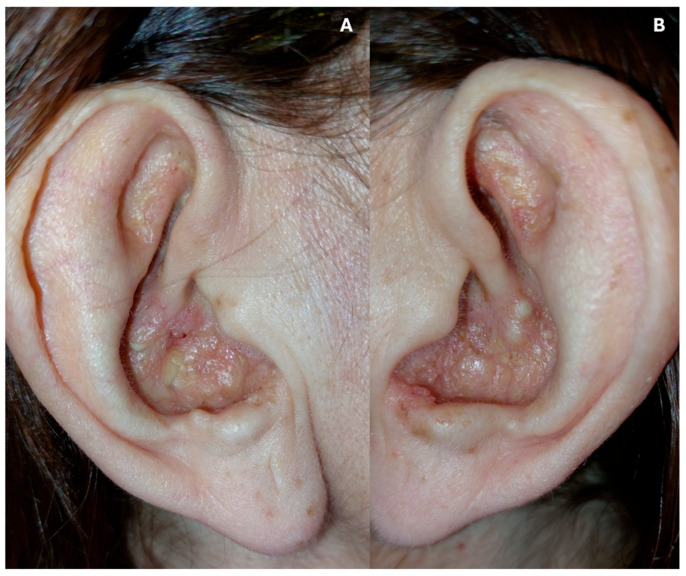
(**A**) Right ear with BLP in remission. (**B**) Left ear with BLP in remission.

Despite these severe gastrointestinal complications, the auricular lesions continued to improve and ultimately showed marked regression under ongoing antibiotic therapy, confirming the inflammatory and non-neoplastic nature of the cutaneous condition (Figure 4).

## 4. Discussion

Blastomycosis-like pyoderma (BLP) remains a significant diagnostic challenge due to its clinical and histopathological overlap with both infectious and neoplastic conditions [1,2,3,4]. The rarity of the disease, combined with its often aggressive and alarming clinical presentation, contributes to frequent misdiagnosis and inappropriate management. In particular, the presence of exuberant vegetative or verrucous plaques in sun-exposed areas may strongly suggest a malignant process, leading clinicians and pathologists toward a diagnosis of squamous cell carcinoma (SCC), especially when evaluating limited or superficial biopsy specimens.

Histologically, BLP is characterized by pseudoepitheliomatous hyperplasia (PEH), which can closely mimic well-differentiated SCC. PEH typically presents with irregular acanthosis, elongation and branching of rete ridges, and a reactive rather than neoplastic epidermal proliferation. In contrast to SCC, cytological atypia is minimal or absent, mitotic figures are rare and normal in appearance, and there is no infiltrative growth pattern breaching the dermoepidermal junction. In our case, epidermal hyperplasia was accompanied by pronounced hyperkeratosis and parakeratosis, and a dense mixed inflammatory infiltrate composed of lymphocytes, histiocytes, neutrophils, and eosinophils. Dilated, keratin-plugged follicles consistent with follicular hypertrophy further supported a reactive inflammatory process.

The histological differential diagnosis of BLP is broad and requires careful clinicopathological correlation. SCC typically exhibits pleomorphic keratinocytes, increased mitotic activity, dyskeratosis, and invasive growth patterns—features that were notably absent in our patient. Verrucous carcinoma, a low-grade variant of SCC, displays broad, pushing rete ridges and keratin-filled crypts but lacks the intense suppurative inflammation and consistent bacterial colonization characteristic of BLP. Deep fungal infections such as blastomycosis and chromoblastomycosis may present with granulomatous inflammation and PEH; however, the identification of fungal elements using PAS (Periodic Acid–Schiff) or GMS (Grocott Methenamine Silver) staining is mandatory for diagnosis and was negative in our case.

Other inflammatory and autoimmune disorders must also be considered. Vegetative pyoderma gangrenosum typically manifests with ulceration, necrosis, and pathergy, rather than hyperplastic epidermal proliferation, and is not associated with persistent bacterial colonization [5]. Pemphigus vegetans presents with vegetating plaques, most commonly in intertriginous areas, and histologically shows acanthosis, eosinophilic abscesses, and intraepidermal acantholysis, accompanied by intercellular IgG deposition on direct immunofluorescence—findings absent in BLP. Rare hypersensitivity reactions, such as iododerma and bromoderma, may also display PEH with eosinophil-rich infiltrates but can usually be distinguished through careful drug history and the absence of identifiable microbial pathogens.

Notably, histological examination showed absence of an irregular deep infiltrative growth pattern, absence of desmoplastic stromal reaction, and preservation of normal epidermal maturation, features that strongly argue against a diagnosis of squamous cell carcinoma.

In our patient, the integration of clinical presentation, histopathological features, and microbiological findings was essential for establishing the correct diagnosis. All diagnostic criteria proposed by Su et al. [6] were fulfilled, including pseudoepitheliomatous hyperplasia with abscess formation, isolation of *Staphylococcus aureus*, and negative fungal and mycobacterial cultures. The unusual bilateral auricular involvement is particularly noteworthy, as auricular localization is rare and bilateral presentation is exceptional, further increasing the likelihood of misdiagnosis as SCC.

From a pathogenetic perspective, BLP is increasingly regarded as a hyperinflammatory cutaneous reaction pattern rather than a true infectious disease. Persistent bacterial colonization, most frequently by *Staphylococcus aureus*, likely acts as a chronic antigenic stimulus. Through the release of exotoxins, superantigens, and other pro-inflammatory mediators, *S. aureus* may drive excessive keratinocyte proliferation and sustained neutrophilic recruitment. This exaggerated inflammatory response results in pseudoepitheliomatous hyperplasia and the characteristic vegetative morphology of the lesions. Importantly, this mechanism may explain why BLP can occur in both immunocompromised and apparently immunocompetent individuals, as subtle immune dysregulation, local barrier dysfunction, or alterations in the cutaneous microbiome may suffice to trigger disease onset.

The auricular region may represent a particularly susceptible site due to chronic environmental exposure, mechanical irritation, and a relatively thin dermis, which may facilitate exaggerated inflammatory responses. In addition, the unique vascular and lymphatic anatomy of the ear may contribute to persistent inflammation and delayed resolution.

An intriguing and clinically relevant aspect of this case is the subsequent development of severe colitis with significant gastrointestinal complications. Although rarely reported, the association between BLP and systemic inflammatory conditions suggests the involvement of shared immunological pathways. Increasing evidence supports the concept of a gut–skin axis, whereby intestinal inflammation, increased gut permeability, microbiota dysbiosis, and systemic cytokine release may influence cutaneous immune responses [1,7,8,9]. In this context, BLP may represent a cutaneous manifestation of systemic immune dysregulation rather than an isolated skin-limited condition. Accordingly, gastrointestinal evaluation should be considered in BLP patients presenting with systemic symptoms or laboratory signs of inflammation.

Our review of 61 previously reported cases [1,2,3,4,5,6,7,8,9,10,11,12,13,14,15,16,17,18,19,20,21,22,23,24,25,26,27,28,29]. (Table 1) highlights several consistent epidemiological trends. BLP predominantly affects middle-aged adults, with a male predominance, but occurs in both immunocompetent and immunosuppressed individuals. Lesions most commonly involve the lower extremities and head and neck region and typically present as vegetative or verrucous plaques, sometimes accompanied by purulent discharge. Histopathologically, pseudoepitheliomatous hyperplasia with intraepidermal and dermal neutrophilic microabscesses is a constant finding, with *Staphylococcus aureus* isolated in approximately 60% of cases.

Timely initiation of culture-guided antibiotic therapy remains the cornerstone of treatment and is associated with complete remission in the majority of patients. Adjunctive therapies, including topical or systemic anti-inflammatory agents, keratinization-modulating drugs such as acitretin, or neutrophil-targeting agents such as dapsone, may be considered in refractory or recurrent cases. Importantly, recognition of BLP as a benign and treatable condition can prevent unnecessary surgical excision and its associated morbidity.

Taken together, these findings underscore the critical importance of careful clinicopathological correlation in differentiating blastomycosis-like pyoderma from its numerous mimickers, particularly squamous cell carcinoma, verrucous carcinoma, and deep fungal infections. A multidisciplinary approach involving dermatologists, dermatopathologists, microbiologists, and, when indicated, gastroenterologists is essential to ensure accurate diagnosis, appropriate therapy, and optimal patient outcomes. In light of these observations, a broader systemic evaluation may be considered in selected patients with blastomycosis-like pyoderma, particularly in cases with extensive, recurrent, or atypical presentations. Such evaluation may include screening for inflammatory bowel disease, hematologic disorders, metabolic diseases such as diabetes mellitus, chronic infections, and other conditions associated with immune dysregulation.

It is important to emphasize that, despite the temporal association observed in this patient, a definitive diagnosis of inflammatory bowel disease could not be established. The gastrointestinal presentation was not typical of classical ulcerative colitis, and therefore no causal relationship between blastomycosis-like pyoderma and colitis can be inferred from this case.

## 5. Conclusions

Blastomycosis-like pyoderma is a rare, benign, and treatable inflammatory dermatosis that can closely mimic malignant and chronic infectious diseases both clinically and histopathologically. Its striking resemblance to squamous cell carcinoma and other hyperplastic or granulomatous conditions represents a major diagnostic pitfall, with potentially significant consequences, including delayed diagnosis, overtreatment, and unnecessary surgical interventions. Accurate recognition of BLP relies on careful integration of clinical findings, histological features, and microbiological data, with particular attention to pseudoepitheliomatous hyperplasia, neutrophilic microabscesses, and bacterial colonization in the absence of true epithelial malignancy.

The present case adds several relevant contributions to the existing literature. First, it confirms that blastomycosis-like pyoderma can occur in apparently immunocompetent individuals, challenging the traditional perception of this condition as being confined to patients with overt immune suppression. This observation supports the hypothesis that subtle immune dysregulation, local barrier impairment, or dysbiosis of the cutaneous microbiome may be sufficient to trigger disease development. Second, the exceptionally rare bilateral auricular involvement expands the known anatomical spectrum of BLP and highlights the importance of considering this diagnosis even in unusual and sun-exposed locations commonly associated with non-melanoma skin cancer.

The favorable clinical response to culture-guided antibiotic therapy further reinforces the inflammatory and non-neoplastic nature of BLP and underscores the importance of conservative therapeutic approaches. Early initiation of appropriate antimicrobial treatment, sometimes combined with topical or systemic anti-inflammatory or keratinization-modulating agents, is associated with excellent outcomes and may prevent unnecessary invasive procedures. In this context, repeated or deeper biopsies, combined with microbiological investigations, should be strongly considered when clinicopathological discordance arises.

An additional noteworthy aspect of this case is the subsequent development of severe colitis, raising the possibility of a link between blastomycosis-like pyoderma and systemic inflammatory conditions. Although such associations are infrequently reported, this observation aligns with emerging evidence supporting the gut–skin axis, whereby intestinal inflammation, microbiota alterations, and systemic immune activation may influence cutaneous inflammatory diseases. From a clinical perspective, this finding suggests that clinicians should maintain a broader diagnostic view and consider evaluation for underlying systemic inflammation in selected patients with BLP, particularly when extracutaneous symptoms or complications are present.

In conclusion, blastomycosis-like pyoderma should be included in the differential diagnosis of vegetative, verrucous, or hyperplastic cutaneous lesions, especially when clinical behavior and histopathological findings are incongruent with malignancy. Increased awareness among dermatologists, dermatopathologists, and surgeons is essential to improve diagnostic accuracy and optimize patient management. Further accumulation of well-documented cases and collaborative studies are needed to better elucidate the pathogenesis of BLP, clarify its relationship with systemic inflammatory disorders, and refine evidence-based diagnostic and therapeutic strategies.

Accordingly, blastomycosis-like pyoderma may represent not only a localized cutaneous reaction but also a potential marker of underlying systemic immune imbalance, warranting a multidisciplinary clinical approach.

## Figures and Tables

**Table 1:** Epidemiology and Clinical Features of Blastomycosis-like Pyoderma.

Parameter	Observation/Summary
Total number of cases	61
Sex distribution	Male predominance (~75%), females ~20%, few unspecified.
Age range (mean)	25–80 years (mean ~50 years). Most cases occur in adults and elderly.
Immune status	Majority immunocompetent (~60%); immunocompromised (~30%), including diabetes, malignancy, or corticosteroid therapy.
Anatomic distribution	
• Head/Neck	Face, scalp, auricular region—frequent localization (≈25%).
• Trunk	Chest, back—uncommon (~10%).
• Upper limbs	Hands and forearms—moderately frequent (~15%).
• Lower limbs	Legs, particularly pretibial and ankle regions—most common (~35%).
• Other/unspecified	Occasional involvement of groin, axillae, perineal area.
Clinical morphology	
• Verrucous or vegetative plaques	Typical presentation in >70% of cases.
• Ulcerated/crusted lesions	~20% of cases.
• Purulent or draining lesions	Occasionally described.
• Pseudoepitheliomatous hyperplasia histologically	Constant finding.
• Single localized lesion	Predominant pattern; multifocal forms rare.
Duration before diagnosis	Ranges from weeks to several years (mean ~8–12 months).
Histopathology	Pseudoepitheliomatous hyperplasia with intraepidermal and dermal neutrophilic abscesses; chronic inflammatory infiltrate.
Microbiology	*Staphylococcus aureus* most frequent (~60%), followed by *Pseudomonas aeruginosa*, *E. coli*, *Morganella morganii*, and mixed infections.

## Data Availability

Data available on request due to restrictions, e.g., privacy or ethical. The data presented in this study are available on request from the corresponding author. The data are not publicly available due to patient privacy.

## Abbreviations

The following abbreviations are used in this manuscript:

BLP	blastomycosis-like pyoderma
SCC	squamous cell carcinoma
PEH	pseudoepitheliomatous hyperplasia
PAS	periodic acid Schiff
GMS	Grocott’s methenamine silver
